# Vinblastine pharmacokinetics in mouse, dog, and human in the context of a physiologically based model incorporating tissue‐specific drug binding, transport, and metabolism

**DOI:** 10.1002/prp2.1052

**Published:** 2023-01-11

**Authors:** Sandra Witta, Keagan P. Collins, Dominique A. Ramirez, Joshua D. Mannheimer, Luke A. Wittenburg, Daniel L. Gustafson

**Affiliations:** ^1^ Flint Animal Cancer Center Colorado State University Fort Collins Colorado USA; ^2^ School of Biomedical Engineering Colorado State University Fort Collins Colorado USA; ^3^ Developmental Therapeutics Program University of Colorado Cancer Center Aurora Colorado USA; ^4^ Department of Surgical and Radiological Sciences University of California Davis California USA; ^5^ University of California Davis Comprehensive Cancer Center Sacramento California USA; ^6^ Department of Clinical Sciences Colorado State University Fort Collins Colorado USA

**Keywords:** comparative oncology, physiologically‐based pharmacokinetic modeling (PBPK), vinblastine

## Abstract

Vinblastine (VBL) is a vinca alkaloid‐class cytotoxic chemotherapeutic that causes microtubule disruption and is typically used to treat hematologic malignancies. VBL is characterized by a narrow therapeutic index, with key dose‐limiting toxicities being myelosuppression and neurotoxicity. Pharmacokinetics (PK) of VBL is primarily driven by ABCB1‐mediated efflux and CYP3A4 metabolism, creating potential for drug–drug interaction. To characterize sources of variability in VBL PK, we developed a physiologically based pharmacokinetic (PBPK) model in Mdr1a/b(−/−) knockout and wild‐type mice by incorporating key drivers of PK, including ABCB1 efflux, CYP3A4 metabolism, and tissue‐specific tubulin binding, and scaled this model to accurately simulate VBL PK in humans and pet dogs. To investigate the capability of the model to capture interindividual variability in clinical data, virtual populations of humans and pet dogs were generated through Monte Carlo simulation of physiologic and biochemical parameters and compared to the clinical PK data. This model provides a foundation for predictive modeling of VBL PK. The base PBPK model can be further improved with supplemental experimental data identifying drug–drug interactions, ABCB1 polymorphisms and expression, and other sources of physiologic or metabolic variability.

## Graphical abstract

Schematic of Vinblastine PBPK Model.

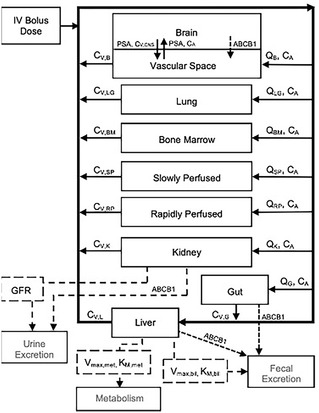

**Abbreviations**
ABCB1: ATP‐binding cassette transporter B1
CYP: cytochrome P450
KO: knock‐out
PBPK: physiologically based pharmacokinetics
PK: pharmacokinetics
VBL: vinblastine
WT: wild‐type

## 1 INTRODUCTION

Vinblastine (VBL) is a vinca alkaloid‐class chemotherapeutic that functions as a microtubule poison. VBL binds to β‐tubulin to destabilize tubulin polymers^1^, ^2^ and prevent further microtubule assembly resulting in mitotic arrest, the induction of apoptosis, and subsequent cell death.^3^, ^4^ VBL has been used successfully to treat both hematologic and solid tumors in both human^5^ and veterinary medicine.^6^ Despite its efficacy, VBL is frequently dose‐reduced due to a variety of toxicities that manifest in hematologic, gastrointestinal, and neurologic pathologies with myelosuppression being most common.^5^ Understanding the physiologic and biochemical components that drive VBL pharmacokinetics (PK) is thus essential to help predict drug exposure, associated toxicities, and reduce related morbidities.

Tissue distribution and intracellular retention of VBL are driven by tubulin binding and interactions with tubulin have been extensively studied.^2^, ^7^ The mechanism underlying VBL‐tubulin binding is a ligand‐induced plus ligand‐mediated isodesmic self‐association reaction, which ultimately results in spiral protofilament structures.^8^, ^9^ Vinca affinity to binding sites present at the terminal ends of microtubules underlies its antimitotic activity as it results in microtubule depolymerization and subsequent metaphase arrest.^10^, ^11^ Tubulin concentrations have been shown to vary substantially by tissue^12^ and the specific β‐tubulin isoform predominantly expressed in cancer can determine vinca alkaloid sensitivity or resistance.^13^

The tissue distribution and elimination of VBL are also influenced by ATP‐binding cassette transporter B1 (ABCB1). ABCB1 expression in normal tissues is primarily in epithelial cells with secretory/excretory functions and endothelial cells of capillary blood vessels serving a barrier function.^14^ This includes the apical surface of endothelial cells of brain capillaries, intestinal and renal tubular epithelial cells, and the canalicular surface of hepatocytes illustrating the role of this protein in drug disposition.^15^ ABCB1 presents a limitation to cellular drug uptake as it functions as an efflux transporter for many lipophilic substrates including VBL.^16^ Previous studies established that wild‐type (WT) mice dosed with VBL showed no brain accumulation, whereas ABCB1 knock‐out (KO) mice (Mdr1a/b(−/−)) accumulated VBL in the brain and gut and showed slower elimination.^17^, ^18^

Metabolism of VBL is primarily attributed to the CYP3A family of enzymes, specifically CYP3A4 and CYP3A12 in humans^19^ and canines,^20^ respectively. The primary metabolite of VBL identified in humans and dogs is the active 4‐deacetylvinblastine and has been reported to have a LD_50_ lower than that of the parent.^21^ As VBL is commonly administered with concomitant medications, drug–drug interactions that potentiate competition for metabolic enzymes through enzyme induction and inhibition are of particular concern when designing VBL therapeutic regimens.

Addressing these variables present a challenge to predicting clinical response but designing physiologically based pharmacokinetic (PBPK) models permits a quantitative approach to reduce the variable complexity and improve understanding of drug‐specific PK.^22^ PBPK models are based on the principle of mass balance and utilize a system of mathematical equations to represent key physiological processes: absorption, distribution, metabolism, and elimination. Kinetic terms characterizing enzymatic reactions that govern metabolism, protein‐facilitated transport of drug, or intracellular binding are preferentially scaled from in vitro data. The concentration–time profiles generated through computational simulation provide a predictive methodology of supplementing traditional noncompartmental analysis by providing a foundation for in vitro‐in vivo (IVIVE) extrapolation, allowing for interspecies scaling, elucidating implications of physiological variability, and ultimately improving drug risk assessment.^23^

Previous human PBPK models have been published for vincristine with incorporation of tubulin as a key driver of drug disposition.^24^, ^25^ These previous models, however, utilized a relative tubulin expression value for all relevant tissues. To our knowledge, the presented model is the first PBPK model of VBL, and the first vinca alkaloid model to incorporate variability in organ tubulin expression and provide an interspecies scaled model from mouse to canine and human. The utility of this model is to illustrate the role of the biophysical drivers of vinca drug disposition with an emphasis on the key tissues at risk of VBL toxicity as well as provide a predictive cross‐species model to enhance dosing strategies.

## 2 MATERIALS AND METHODS

### 2.1 Chemicals

Vinblastine sulfate salt and vinorelbine ditartrate salt hydrate were purchased from Sigma‐Aldrich. Vinblastine (for injection) and vincristine were obtained from the pharmacy at the Colorado State University Veterinary Teaching Hospital. 4‐desacetylvinblastine was purchased from Santa Cruz Biotechnology. Acetonitrile and methanol used for LC–MS/MS were of ULPC/MS grade and were purchased from Fisher Scientific. All other reagents were of analytical grade and were purchased from commercial suppliers.

### 2.2 VBL PK study in mice and sample preparation

Mouse protocols were approved by the Institutional Animal Care and Use Committee at Colorado State University. Two strains of mice were used: FVB female mice and Mdr1a/b (−/−) (knock‐out, KO) female mice in a FVB background (Taconic Biosciences, Inc) for a total of 15 mice per strain with three mice per time point. Mice were dosed IV with 2 mg/kg VBL prepared in 0.9% benzyl alcohol. Whole blood and tissues were harvested throughout a time range of 0.08 to 6 h. Whole blood was collected with heparinized needles and transferred to a microcentrifuge tube and was spun at 1200x*g* for 10 min at 4°C. The resulting serum and tissues were flash‐frozen in liquid nitrogen and stored at −80°C until ready for analysis.

### 2.3 VBL PK study in dogs

A prospective clinical trial was performed at Colorado State University Veterinary Teaching Hospital. Prior to starting the clinical trial, the protocol was approved by the Colorado State University Animal Care and Use Committee. Written informed consent was obtained from all owners before treatment started. Dogs enrolled in this study consisted of client‐owned dogs with cytologically or histologically confirmed MCT whose owners elected to pursue vinblastine chemotherapy. Inclusion criteria were body weight > 10 kg, adequate blood work (total bilirubin not exceeding 1.5× normal; transaminases not exceeding 2× normal; creatinine not exceeding 2× normal; at least 2500 neutrophils/μl, 75 000 platelets/μl, and a hematocrit of at least 28%), a VCOG performance status of 0 or 1 (0, normal activity; 1 restricted [decreased activity from pre‐disease status]; 2, compromised [ambulatory for only vital activities, urinates and defecates in appropriate areas]; 3, disabled [requires force feeding, unable to urinate and defecate in appropriate areas]; 4, dead). No prior vinblastine was allowed and a 2‐week washout period from surgery, other chemotherapies or kinase inhibitors was required. Concurrent prednisolone, gastroprotectants, and diphenhydramine were allowed. Dogs were treated at Colorado State University Veterinary Teaching Hospital between March 2011 and July 2012. Dogs received vinblastine sulfate at 2.5 mg/m^2^ as an IV bolus. Serum was collected at 0, 5, 10, 15, 30, 45, 60, 90, 120, 240, 360, 480, 720, and 1440 min following treatment.

### 2.4 Microsomal metabolism

Microsomes were purchased from XenoTech, LLC. Mouse microsomes (M1500, batch: 1410027) were derived from female CD‐1 mice of mixed age. Canine microsomes (D1500, batch: 1310105) were derived from purpose‐bred female beagles of mixed age from 4 to 9 years.

Microsome incubation and Michaelis–Menten kinetics experiments were modified from previously published methods.^26^ Microsome incubation reactions for VBL were conducted at a time range from 0 to 45 min with a starting substrate concentration of 1 μg/ml for both mouse and canine microsomes. Incubation reactions were performed in technical singlet. Michaelis–Menten kinetics experiments were performed following a discontinuous methodology using the following parameters, determined from the microsome incubations: mouse VBL (0.5 mg/ml protein, 5 min); canine VBL (0.5 mg/ml, 20 min). These conditions were chosen as they represented linear loss of parent with respect to time, satisfying the steady‐state assumptions for Michaelis–Menten kinetics analysis. Kinetics experiments were performed in technical doublet. Both microsome incubation and kinetics reactions were stopped following the same method and analyzed by LC–MS/MS using the described method. Loss of parent was quantified absolutely and converted to velocity (ng/ml/min) and used as a surrogate for product formation. Michaelis–Menten kinetics parameters were estimated from velocity versus substrate data using GraphPad Prism v7.0d (GraphPad Software Inc).

### 2.5 Sample preparation for LC/MS/MS analysis

Serum samples (200μl) were spiked with 50 ng (10 μl of 5 μg/ml solution) of vincristine as an internal standard. Twenty microliters of vinblastine (or 4‐deacetylvinblastine) standard (or blank diluent for unknowns) was then added to standard and quality control samples at a range of 1–1000 ng/ml final concentration, vortexed for 10 min, and loaded onto solid phase extraction cartridges (Oasis® HLB; 1 cc, 30 mg; Waters Corporation). During vortex mixing, solid‐phase extraction cartridges were prepared by the addition of 400 μl methanol followed by 400 μl milli‐Q water. Loaded samples were then washed twice with 5% methanol. Vinblastine, 4‐deacetylvinblastine, and vincristine were then eluted from the column with 400 μl of methanol into glass tubes. Samples were then evaporated to dryness using a Savant AES 1000 SpeedVac. Samples were then resuspended in 100 μl of diluent and transferred to autosampler vials with glass inserts for injection onto the HPLC system.

### 2.6 LC/MS/MS instrumentation and conditions

Positive ion electrospray ionization (ESI) mass spectra were obtained with a MDS Sciex 3200 Q‐TRAP triple quadrupole mass spectrometer (Applied Biosystems Inc) with a turbo ionspray source interfaced to a Shimadzu HPLC system and an HTC‐PAL autosampler (Leap Technologies). Samples were chromatographed with an Agilent ZORBAX Rx/SB‐C 8 (4.6 × 75 mm) column (Agilent Technologies) protected by a C 18 guard cartridge, 4.0 mm by 2.0 mm (Phenomenex). An LC gradient was employed with mobile phase A consisting of 10 mM ammonium acetate pH 4.0 and mobile phase B consisting of acetonitrile. Chromatographic resolution was obtained by holding mobile phase B steady at 50% from 0 to 0.01 min, increasing linearly from 50% to 90% from 0.01 to 3.0 min, holding steady at 99% from 3.0 to 4.5 min, decreasing linearly from 99% to 50% from 4.5 to 5.0 min, followed by equilibration at 50% from 5.0 to 6.0 min. The LC flow rate was 0.75 ml/min and the sample injection volume was 10 μl. The analysis run time was 6.0 min.

The mass spectrometer settings were optimized for vinblastine as follows: turbo ionspray temperature (T), 400°C; ionspray voltage, 5000 V; declustering potential (DP), 41 V; entrance potential (EP), 10 V; collision energy (CE), 59 V; collision cell entrance potential (CEP), 25 V; collision cell exit potential (CXP), 4.0 V; curtain gas, N 2, (CUR), 30 units; collision gas, N 2, (CAD), medium; nebulizer gas, N 2, 40 units; and auxiliary gas, N 2, 60 units. The predominant product ions for vinblastine, 4‐desacetylvinblastine, and vincristine were m/z 811.5, 769.4, and 825.4, respectively.

Samples were quantified by internal standard reference method in multiple reaction monitoring (MRM) mode monitoring ion transitions m/z 811.5 → 224.3 for vinblastine, m/z 769.4 → 355.2 for 4‐desacetylvinblastine, and m/z 825.4 → 765.3 for vincristine (IS). The dwell times for each ion transition were 500 ms. Q1 and Q3 were both operated in unit resolution. Column retention times were 2.83 min for vinblastine, 2.36 min for vincristine, and 1.80 min for 4‐desacetylvinblastine. No interfering peaks were detected at the monitored ion transitions in extracted matrix.

### 2.7 Data analysis

Quantitation of vinblastine and its metabolite was based on linear standard curves in spiked matrix (serum) using the ratio of vinblastine or metabolite peak area to vincristine peak area and 1/x^2^ weighting of linear regression. Analyst v1.5.1 software (Applied Biosystems) was used for peak area integration. For serum samples, the lower limit of vinblastine detection was 1 ng/ml and the curve was linear from 1 to 750 ng/ml. For 4‐desacetylvinblastine, the lower limit of quantitation was 1 ng/ml and the curve was linear from 1 to 100 ng/ml. All calculated concentrations from unknown samples fell within the linear range of the respective standard curves. Accuracy of standard curves and quality control samples (low, medium, and high concentrations) were within 15% for vinblastine and 10% for 4‐desacetylvinblastine and >80% of quality control samples showed an accuracy >85%.

### 2.8 PBPK model development

**FIGURE 1:**
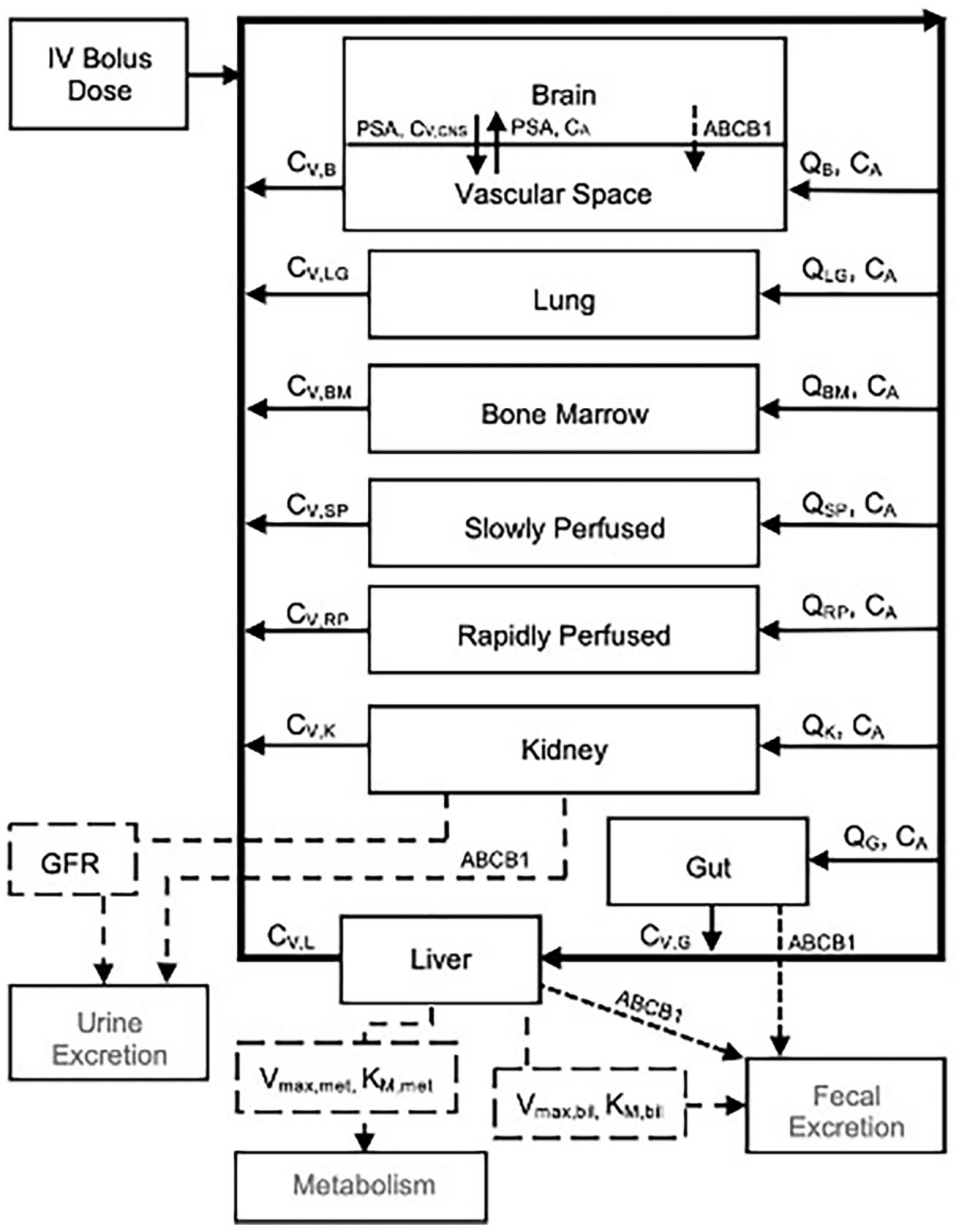
VBL PBPK model schematic. Schematic representation of a physiologically based pharmacokinetic (PBPK) model including key organs involved in vinca drug ADME following a bolus IV dose. Solid lines are representative of blood flows and dashed lines represent clearance from organs by ABCB1‐mediated transport, metabolism (V_max,met_ and K_m,met_), biliary excretion (V_max,bil_ and K_m,bil_), or glomerular filtration (GFR).

The PBPK model for VBL, represented by the schematic in Figure 1 is characterized by a set of nine distinct tissue compartments: plasma, brain, lung, bone marrow, slowly perfused, rapidly perfused, kidney, liver, and gut. The model's objective is to provide a unique and predictive, cross‐species modality of describing the absorption, distribution, metabolism, and elimination of VBL. This model thus aims to elucidate the implications of drug pharmacokinetics and mechanisms of distribution, ultimately providing a modeling base for extrapolation of simulated concentration profiles to clinical settings. The primary drivers of vinca disposition are fraction unbound drug to plasma proteins, intercellular tubulin binding, metabolism, and ABCB1‐mediated transport. Each tissue is characterized by its corresponding volume and flow rate indicated in Table 1. With exception to the brain, tissue compartments are defined by a well‐stirred, flow‐limited model in which the concentration of drug leaving the compartment is equal to the unbound drug concentration within the tissue. The blood–brain barrier (BBB) is distinctive as the tight junctions and prolific expression of efflux transporter proteins provide a highly restrictive barrier to many lipophilic molecules. The brain is thus defined by a permeability‐limited model dictated by a modification of Fick's Law of diffusion in which the permeability‐surface area product constitutes the tissue permeation coefficient, the capillary surface area permitting diffusion, and the thickness of the cellular membrane.^32^ ABCB1 is highly expressed at the blood–brain barrier as well as in the liver, gut, and kidneys, facilitating drug excretion.^14^, ^15^

**TABLE 1:** PBPK model parameters used in model simulations.

	Mouse	Dog	Human
% Body weight^a^			
Brain	1.65	0.78	2
Bone marrow^b^	3.1	1.2	1.1
Kidney	1.67	0.5	0.44
Liver	5.49	3.3	2.57
Gut	4.22	3.68	2.83
Slowly perfused	70.5	75.8	77.3
Rapidly perfused	8.43	6.53	5.86
Blood	4.9	8.2	7.9
% Cardiac output^a^			
Brain	3.3	2	11.4
Bone marrow^b^	1	3	3
Kidney	9.1	17.3	17.5
Liver	2	4.6	4.6
Gut	13	25.1	18.1
Slowly perfused	35	38	34.3
Rapidly perfused	36.6	10	11.1
Partition coefficients^c^			
PC_Gut_	1.08		
PC_Kidney_	3.73		
PC_Liver_	2.26		
PC_BM_	1.0		
PC_CNS_	1.21		
PSA [ml/h]^c^	0.379		
PC_blood_ ^e^	1.0		
Protein binding^d^	48–99%; 75%		
HCT^d^	0.42–0.45		

^a^ Sourced from Brown et al.^27^

^b^ Bone marrow blood flow in mice was estimated from rat data.^28^ Canine and human blood flow values were estimated from human data.^29^

^c^ Partition coefficients and permeability surface area (PSA) of the blood–brain barrier optimized to mouse mdr1a/b (−/−) data.

^d^ Sourced from published values.^5^, ^30^, ^31^

^e^ Bone marrow body weight percent determined using an allometric scaling equation as a function of body weight for rats.^27^

### 2.9 Model parameterization & equations

Physiological parameters for tissue‐specific percent body weight and cardiac output for mouse, canine, and human were sourced from Brown et al.^27^ and presented in Table 1. All tissue densities were approximated to be 1 kg/L. Cardiac output for respected species was estimated using a previously derived allometric scaling relationship for unanesthetized mice.^33^ The percentage of drug bound to plasma proteins, primarily alpha‐1 acid glycoprotein, is 48–99% with as much as 75% bound to serum proteins in dog plasma.^5^, ^30^ The rate of change in the amount of drug within generic, non‐eliminating compartments is governed by the following mass balance equation:

(1) $$\frac{dA_{C}}{\mathrm{dt}}=\frac{V_{C}\times dC_{C}}{\mathrm{dt}}=Q_{C}\times \left(C_{A}-C_{\mathrm{VT}}\right)$$

where $A_{C}$ is the amount of drug in the tissue, $V_{C}$ is the volume of the tissue (assuming static volume), $C_{C}$ is the concentration within the tissue, $Q_{C}$ is the tissue perfusion rate, $C_{A}$ is the arterial blood drug concentration, and $C_{\mathrm{VT}}$ is the venous blood drug concentration.

A key mechanistic feature of this model is the tissue‐specific intracellular binding of VBL to tubulin. The variability in tubulin‐binding capacity of different tissues has been shown to have a deterministic role in vinca alkaloid tissue distribution.^12^ To account for intracellular VBL retention in all tissues incorporated into the PBPK model, the following equation was used to determine the venous blood drug concentration:

(2) $$\;C_{\mathrm{VT}}=\frac{C_{T}}{\left(P_{C}\right)+\left(\frac{B_{C}}{K_{D}+C_{\mathrm{VT}}}\right)}\;$$

where $C_{T}$ is the concentration of drug in the compartment, $P_{C}$ is the partition coefficient, $B_{C}$ is intracellular‐binding capacity of drug to tubulin, and $K_{D}$ is the drug‐specific‐binding affinity to tubulin. The use of a nonlinear mathematical equation to characterize drug binding to intracellular macromolecules has been reported in describing the disposition of docetaxel^34^ and methotrexate^22^ as a function of binding at low drug concentrations. Tissue‐specific‐binding capacities, $B_{C}$, were obtained from studies where tubulin concentrations were determined from tubulin‐binding capacities for colchicine^12^ and are reported in Table 2. A strong correlation between tissue‐to‐plasma partition coefficients and binding capacity for the vinca alkaloid, vincristine, has been reported for mouse, rat, dog, and monkey.^12^ Relative tissue tubulin‐binding capacities were thus assumed to be equivalent for the mouse, dog, and human PBPK model developed herein. The intrinsic value for VBL‐binding affinity for tubulin, $K_{D}$, is reported in Table 2 as 196 nmol/L. The binding affinity report was measured using a ligand‐mediated model where the affinity for liganded heterodimers for spiral polymers was found to be the major determinant of overall vinca drug affinity to tubulin.^35^ Partition coefficients, $P_{C}$, for respective tissues were determined by performing parameter optimization in MATLAB's tool, SimBiology, using a nonlinear least squares regression estimation method from the optimization toolbox. The statistical modeling method fit pooled Mdr1a/b (−/−) tissue data generating one set of partition parameters for all tissues which are reported in Table 1.

**TABLE 2:** Tubulin & ABCB1 expression parameters.

Tissue‐specific tubulin‐binding capacity [nmol/kg]^a^	
Brain	10 710 ± 1320
Lung	2580 ± 390
Kidney	1470 ± 190
Liver	3510 ± 290
Gut	1080 ± 160
Muscle	900 ± 130
Rapidly perfused	3420 ± 190
Slowly perfused	900 ± 130
Bone marrow^b^	371.25
Kd [nM]^c^	196.08
ABCB1 expression scaling factors^d^	
SF_brain_	1.0
SF_gut_	0.14
SF_liv_	0.28
SF_kid_	0.78

^a^ Tubulin‐binding capacity expressed as mean ± SD as previously reported.^12^

^b^ Bone marrow tubulin‐binding capacity optimized to mdra1a/b (−/−) data.

^c^ VBL‐binding affinity for tubulin sourced from Lobert et al., 1996.^35^

^d^ Tissue‐specific scaling factors for ABCB1 protein expression sourced from Systems Pharmacology for ABCB1 relative tissue expression as reported.^36^

The rate of change in the amount of drug in tissues with ABCB1 expression is as follows:

(3) $$\;\frac{dA_{T}}{\mathrm{dt}}=Q_{C}\times \left(C_{A}-C_{\mathrm{VT}}\right)-F_{\text{ABCB}1,T}\times \left(\frac{\;V_{\max,\text{ABCB}1}\times C_{v}}{K_{m}+C_{v}}\right)\;$$

In which ABCB1‐mediated efflux of drug is characterized by Michaelis–Menten saturable kinetics, where $F_{\text{ABCB}1,T}\;$ is the scaling factor for the relative expression of ABCB1 transport protein in relevant tissues, $V_{\max,\text{ABCB}1}$ is the maximum velocity of ABCB1 transport out of the tissue compartment, and $K_{m}$ is the associated Michaelis–Menten constant. Scaling factors, $F_{\text{ABCB}1,T}$, were acquired from Systems Pharmacology for ABCB1 relative tissue expression as previously reported.^36^

The transfer of drugs at the BBB is unique as the mass transfer out of the vascular space surrounding the brain tissue is hindered by a permeability barrier. The highly restrictive nature of the BBB to exogenous substances results in a diminished flux of drug into the tissue, warranting the use of a permeability‐limited modeling approach.^32^ Coupling the ABCB1 transport equations as expressed above with permeability‐limited modeling equations from Choi et al.,^23^ the following equations are proposed where Equation (4) describes the amount of drug within the brain tissue and Equation (5) describes the amount of drug in the vascular space surrounding the tissue:

(4) $$\begin{array}{c} \;V_{\mathrm{CNS}}*\left(\frac{dC_{\mathrm{CNS},T}}{\mathrm{dt}}\right)=\mathrm{PSA}\times \left(C_{A}-C_{\mathrm{CNS},V}\right)\\ \;-sf_{\text{ABCB}1,\mathrm{CNS}}\times V_{\mathrm{CNS}}\times \left(\frac{V_{{\mathrm{MAX}}_{\text{ABCB}1}}\times C_{\mathrm{CNS},V}}{K_{M_{\text{ABCB}1}}+C_{\mathrm{CNS},V}}\right)\; \end{array}$$

(5) $$\begin{array}{c} V_{\mathrm{BB}}*\left(\frac{dC_{\mathrm{BB}}}{\mathrm{dt}}\right)=Q_{\mathrm{CNS}}*\left(C_{A}-C_{V}\right)-\mathrm{PSA}\times \left(C_{A}-C_{V,\mathrm{CNA}}\right)\\ \;+sf_{\mathrm{CNS}}\times V_{\mathrm{CNS}}\times \left(\frac{V_{{\mathrm{MAX}}_{\text{ABCB}1}}\times C_{\mathrm{CNS},V}}{K_{M_{\text{ABCB}1}}+C_{\mathrm{CNS},V}}\right)\; \end{array}$$

where $V_{\mathrm{CNS}}\;$ is the volume of the brain tissue, $V_{\mathrm{BB}}\;$ is the volume consisting of brain blood, $C_{\mathrm{CNS},T}$ is the concentration of drug in the brain tissue, PSA is the permeability surface area product, $sf_{\text{ABCB}1,\mathrm{CNS}}$ is the relative ABCB1 expression in brain tissue, and $C_{\mathrm{BB}}$ is the concentration of drug in the brain blood. $V_{\mathrm{CNA}}$ and $V_{\mathrm{BB}}$ were set to 97% and 3% of total brain volume, respectively.^37^ The PSA value representing the flux of VBL at the BBB was determined by optimizing the simulated Mdr1a/b(−/−) brain concentration versus time profile with in vivo VBL Mdr1a/b(−/−) mouse brain data. The parameter optimization method was conducted as described previously with nonlinear least squares regression. The calculated PSA value of 0.376 ml/h, as reported in Table 1, characterizes the baseline flux of VBL at the BBB in the absence of ABCB1‐mediated transport and was used in subsequent dog and human models. $V_{\max}$ and $K_{m}$ associated with ABCB1‐driven transport were simultaneously fit to wild‐type brain tissue PK data and determined to be 928.8 nM/h and 6.41 nM as reported in Table 3. Extrapolation of ABCB1 kinetic parameters to the mouse and human models was conducted using a K_m_ value set to 5.76 μM as reported in literature for human ABCB1 ATPase activity for VBL.^40^ The corresponding $V_{\max,\text{ABCB}1}$ value was optimized to in vivo canine PK data. The analyzed PK data for VBL‐treated canines included the following concurrent treatments groups: prednisone, omeprazole, diphenhydramine (n = 8), prednisolone (n = 4), and no concurrent medications (n = 1) as shown in Figure 3. Omeprazole has been shown to be an inducer of CYP3A4 in human hepatocytes^41^ and an inhibitor of ABCB1 in Caco‐2 cell lines.^42^ Canine model parameter values were thus fit to the patients without concurrent treatment of omeprazole to minimize the influence of potential drug–drug interactions.

**TABLE 3:** Drug clearance parameters for metabolism, ABCB1‐mediated transport, and excretion.

	Mouse		Dog, Human
Metabolism^a^, ^b^			
Vm_met_ [umol/L/h]	1794.9		189.6
Km_met_ [umol/L]	11.6		9.53
ABCB1^c^			
Vm_ABCB1_ [nmol/L/h]	928.8		64.54
Km_ABCB1_ [nmol/L]^d^	6.41		5760
Biliary transport^c^	mdra1a/b (−/−)	WT	
Vm_bil_ [nmol/L/h]	23.35	928.8	64.54
Km_bil_ [pmol/L]	0.0152	6.41	5760
GFR	0.11		

^a^ Michaelis–Menten parameters for mouse metabolism determined experimentally using mouse microsomes (0.5 mg/ml). Kinetic parameters extrapolated using 46 mg protein/g liver for in vitro‐in vivo scaling.^38^

^b^ Michaelis–Menten parameters used in dog and human models sourced from experimental studies using dog liver microsomes.^20^ Kinetic parameters estimated using nonlinear regression fitting of Web‐Plot digitized data and extrapolated using 55 mg protein/g liver for in vitro‐in vivo scaling.^39^

^c^ Optimized Michaelis–Menten parameters for ABCB1 activity and biliary excretion.

^d^ Km for Dog and Human ABCB1 transport based on ATPase activity.^40^

The primary routes of vinca drug elimination are through metabolism, biliary excretion, and glomerular filtration. Clearance from the gut, kidneys, and liver is also mediated by ABCB1 as it contributes to tubular secretion and biliary excretion.^15^ Previous studies have shown a decrease in fecal excretion from 20%–25% in wild‐type mice to 3%–9% in Mdr1a/b (−/−) mice at doses of 1 and 6 mg/kg.^18^ To account for drug elimination, additional terms were added to Equation (3). Metabolism is represented by Equation (6), biliary clearance is represented in Equation (7), and glomerular filtration is modeled using Equation (8).

(6) $$\;\frac{\mathrm{dC}L_{\text{Metabolism}}}{\mathrm{dt}}=V_{\mathrm{LIV}}\times \left(\frac{V_{\max,M}\times C_{V}}{K_{m,M}+C_{V}}\right)$$

(7) $$\;\;\frac{\mathrm{dC}L_{\text{Biliary}}}{\mathrm{dt}}=V_{\mathrm{LIV}}\times \left(\frac{V_{\max,B}\times C_{V}\;}{K_{m,B}+C_{V}}\right)$$

(8) $$\;\frac{\mathrm{dC}L_{\text{Filtration}}}{\mathrm{dt}}=\mathrm{GFR}\times Q_{\mathrm{KID}}\times C_{A}$$

Metabolism and biliary transport were modeled with saturation kinetics. Metabolic kinetic parameters for the mouse model were derived experimentally using mouse microsomes of 0.5 mg/ml and extrapolated using 46 mg protein/g liver for in vitro‐in vivo scaling.^38^ Metabolic kinetic parameters were estimated from fitting Web‐plot digitized data from canine liver microsmal studies by^20^ and extrapolated using 55 mg protein/g liver for IVIVE.^39^ Scaling of metabolic $V_{\max}$ was determined using the following equation as reported previously^26^:

$$V_{\max,M}=V_{\max,\mathrm{mic}}*P_{\mathrm{mic}}*V_{\mathrm{LIV}}$$

where $V_{\max,\mathrm{mic}}$ is the Michaelis–Menten rate constant determined experimentally using liver microsomes and $P_{\mathrm{mic}}$ is the micoromal protein per gram of liver. Values for species‐specific kinetic parameters are reported in Table 3. Biliary excretion in the mouse model was developed using baseline biliary excretion parameters, $V_{\max,B}$ and $K_{m,B}$, through the previously described optimization method to Mdr1a/b (−/−) fecal data as shown in Figure 1G. The presence of VBL in fecal data of Mdr1a/b (−/−) is assumed to be attributed to Mrp2/ABCC2 present on canalicular cells of hepatocytes in rodents^43^ as VBL has been identified as a substrate for human MRP2/ABCC2.^44^ Biliary excretion was assumed to be primarily driven by ABCB1‐mediated transport in the wild‐type mouse, canine, and human models. Biliary transport kinetic values were thus equivalent to those of ABCB1 as reported in Table 3 and scaled to the liver using the tissue‐specific relative expression scaling factor indicated in Table 2.

### 2.10 Computer simulation and software

The PBPK model and simulations were conducted using SimBiology (MATLAB, version r2021b) from MathWorks Inc. Parameter optimization was generated through MATLAB's Optimization Toolbox, using a pooled dataset fit and nonlinear least‐squares solver. The validity of parameter optimizations was evaluated using a combination of residuals, Akaike's information criterion, and Schwarz information criterion.

Monte Carlo simulations of physiologic parameters were performed using the SimBiology Model Analyzer app to generate virtual patients. Sample parameter values were drawn from a lognormal distribution with the standard deviation of logarithmic values set to 0.2 for parameters lacking literature‐reported standard deviations. The sampling of values was constructed using random sampling with rank correlation matrix.

### 2.11 Pharmacokinetic analysis

Pharmacokinetic parameters were calculated using Phoenix WinNonlin, version 8.3, Certara. Area under the curve (AUC) was calculated using a linear‐log trapezoidal method.

### 2.12 Data analysis

The predictive capability of the model was evaluated by calculating the prediction error (PE), median absolute performance error (MAPE %), the median performance error (MPE %), and the root mean squared performance error (RMSPE %) as indicated in Equations (9), (10), (11), (12).^45^ The prediction was calculated as follows:

(9) $$\mathrm{PE}=\frac{C_{\text{measured}}-C_{\text{predicted}}}{C_{\text{predicted}}}\times 100\%$$

The measure of the precision of the prediction, evaluated was evaluated by the MAPE % and calculated with Equation 10 where *n* is the total number of samples per tissue.

(10) $$\;\text{MAPE}\%=\text{median}\;\left(\left|PE_{1}\right|,\left|PE_{2}\right|,\ldots \left|PE_{n}\right|\right)$$

The bias of the prediction was calculated by MPE% as:

(11) $$\mathrm{MPE}\%=\text{median}\;\left(PE_{1},PE_{2},\ldots PE_{n}\right)$$

The accuracy of the prediction was calculated using RMPSE % as:

(12) $$\;\text{RMSPE}\%=\sqrt{\frac{\sum_{i=1}^{n}PE_{i}^{2}}{n}}$$

## 3 RESULTS

### 3.1 VBL PK and model simulations in mice

VBL plasma and tissue concentrations were determined in Mdr1a/b (−/−) mice and wild‐type mice after a single IV bolus dose of 2 mg/kg. Plasma and tissue concentrations were measured at time intervals between 0.83 and 6 h post‐drug administration. The mouse PBPK model for Mdr1a/b (−/−) mice and wild‐type mice was developed using SimBiology, MATLAB with a focus on the following tissues: plasma, brain, bone marrow, liver, gut, and kidney. The primary drivers of vinca disposition including tubulin binding, ABCB1‐mediated transport, and elimination via metabolism, biliary clearance, and glomerular filtration created the foundation of the model. Metabolism values were either derived experimentally or sourced from literature, while tissue partitioning and Michaelis–Menten parameters for ABCB1 transport and biliary excretion were optimized using plasma and tissue PK data.

**FIGURE 2:**
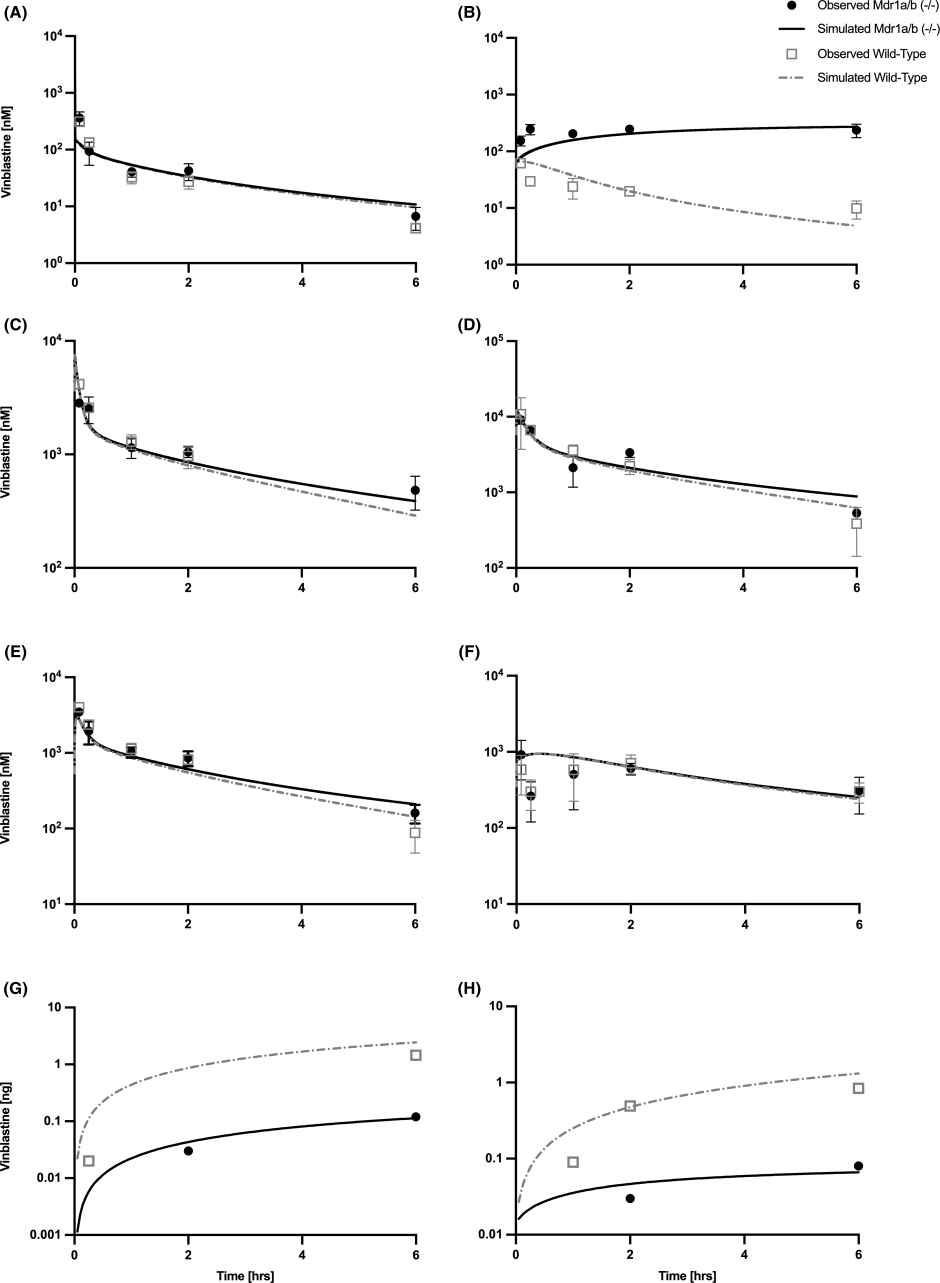
Observed and model simulated vinblastine concentrations in mouse plasma (A), brain (B), gut (C), kidney (D), liver (E), bone marrow (F), feces (G), and urine (H) for a dose of 2 mg/kg in wild‐type and Mdr1a/b(−/−) mice (*n* = 3). Symbols represent measured values and the lines PBPK model simulations. Wild‐Type mice, open squares; Mdr1a/b(−/−) mice, filled circle; Wild‐Type simulation, dashed line; Mdr1a/b(−/−) simulation, solid line. All observed data are shown as mean ± SD.

The resulting PBPK model simulations for both wild‐type and Mdr1a/b (−/−) mice are shown in Figure 2 and well predict the observed PK data. The role of ABCB1 is most prominently shown in the brain PK and simulations in Figure 2B where the wild‐type profile indicates a gradual decrease in VBL tissue concentration in contrast to the steady accumulation of drug in brain tissue of Mdr1a/b (−/−) mice. Tissue‐specific characteristics governing disposition and clearance were successfully captured by the PBPK model as shown by the concentration–time profiles. Bone marrow is unique as it has distinctly greater retention of VBL than other relevant tissues as represented by the in vivo data and simulations in Figure 2F.

Serum and tissue PK parameters, area under the curve (AUC), and terminal half‐life were calculated for both observed and simulated data as reported in Table 4. Ratios of observed versus simulated PK parameters for corresponding tissues were calculated to provide a direct comparison. The results showed that the predicted AUC ratios were within the accepted validating criteria of twofold with the largest discrepancy of 78% for the gut.^46^ The ratios for terminal half‐life for serum and tissues were predominantly within the range of twofold, apart from wild‐type brain and gut with values of 0.45 and 2.07, respectively.

**TABLE 4:** Pharmacokinetic parameters from mouse wild‐type and Mdr1a/b (−/−) VBL observed (Obs) data and PBPK model simulations (Sim).

	AUC_0‐6h_ (nM × h)^a^			t_1/2_ (h)^b^		
	Obs	Sim	Ratio^c^	Obs	Sim	Ratio^c^
Wild‐type mice						
Serum	181	195	1.08	1.60	2.09	1.3
Brain	109	121	1.12	3.94	1.76	0.45
Gut	3467	4613	1.33	1.27	2.62	2.07
Kidney	12 627	11 816	0.94	1.56	2.32	1.48
Liver	4212	3299	0.78	1.32	1.96	1.49
Bone marrow	2983	3194	1.07	NA^d^	2.77	NA^d^
Mdr1a/b (−/−) Mice						
Serum	215	201	0.94	1.62	2.22	1.37
Brain	1401	1247	0.89	NA^d^	NA^d^	NA^d^
Gut	5896	5052	0.86	3.86	3.28	0.85
Kidney	13 462	13 066	0.97	1.79	2.92	1.63
Liver	4268	3674	0.86	1.80	2.43	1.35
Bone marrow	2712	3259	1.20	5.83	2.91	0.49

^a^ AUC_0–6 h_ is the area under the concentration–time curve profile from 0 to 6 h.

^b^ t_1/2_ is the terminal half‐life as calculated from linear regression of the terminal elimination phase.

^c^ Ratio represents Sim value divided by the Obs value.

^d^ Terminal elimination phase was not achieved.

The predictive performance of the PBPK model was evaluated by calculating MAPE%, MPE%, and RMSPE % as reported in Table 7. The accuracy of the prediction, measured by MAPE%, ranged between 19.3%‐42.3% and 20.9%–30.0% for wild‐type and Mdr1a/b (−/−), respectively. The MPE% is a method of evaluating the models under‐ or overprediction which indicated a lack of consistent bias as three of the seven tissues showed negative MPE% for both wild‐type and Mdr1a/b (−/−) models.

### 3.2 VBL PK and model simulations in canines

After validating the accuracy of the above PBPK model, the wild‐type mouse model was scaled to canine using the physiological parameters presented in Table 1. and clearance parameters indicated in Table 3. A Monte Carlo simulation was then performed with standard deviations corresponding to experimentally determined parameter values or 0.2 for parameters lacking standard deviations. A virtual population of 100 was generated using random sampling with a rank correlation matrix.

Time course serum samples were collected from a clinical trial performed at Colorado State University Veterinary Teaching Hospital for canine patients undergoing VBL chemotherapy. Patients were either on no reported concomitant medications, prednisolone, or prednisone, omeprazole, and diphenhydramine (POD).

**FIGURE 3:**
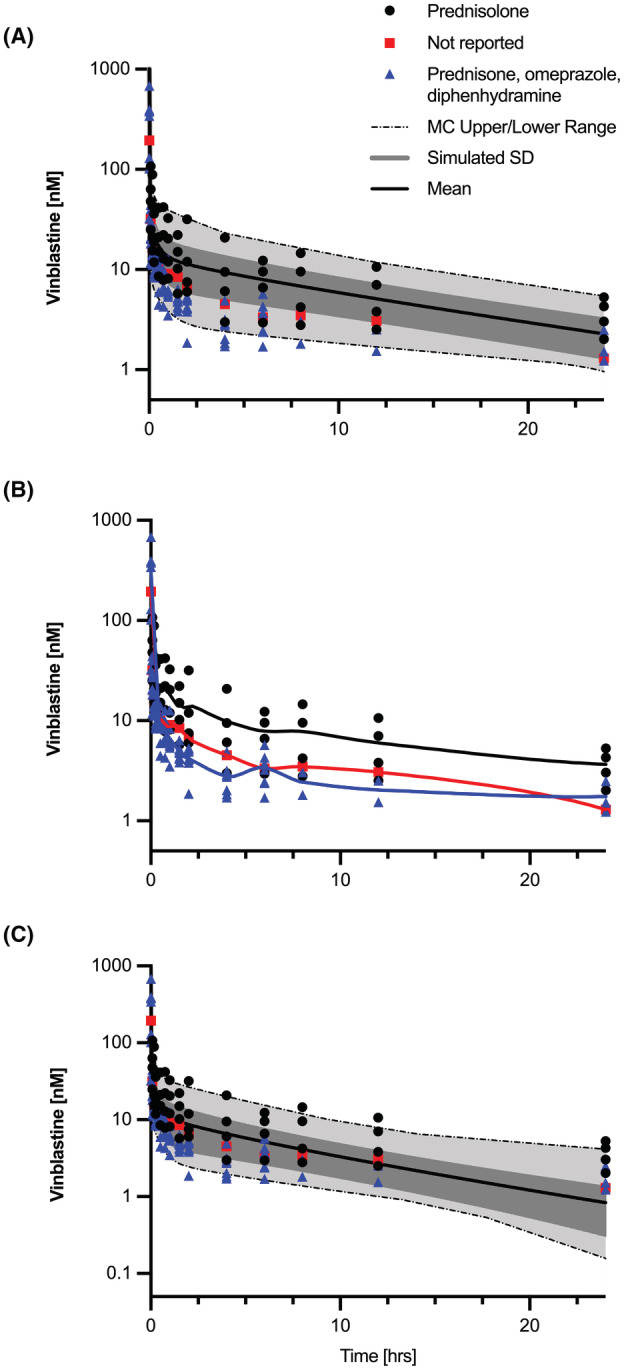
Observed canine plasma PK with indicated patient concomitant medication treatment groups: prednisolone only (*n* = 4), not reported (*n* = 1), and prednisone/omeprazole/diphenhydramine (*n* = 8); Monte Carlo PBPK model simulations (*n* = 100) are represented by range, SD, and mean outputs (A). Observed canine plasma PK with an Akima spline fit to concomitant medication treatment groups (B). Monte Carlo simulation of canine plasma PK following an induction in metabolism due to omeprazole‐induced increase in CYP3A4 mRNA expression by twofold as determined^41^ (C).

The PBPK model simulations and PK data are presented in Figure 3. An Akima spline fit for each treatment group in Figure 3B highlights the discrepancy between the concentration versus time profiles between the prednisolone and the POD treatment groups. AUC and terminal half‐lives were determined for mean simulated and PK data based on indicated concomitant treatment groups presented in Table 5. Ratios of observed versus simulated AUC's and terminal half‐lives were determined. For all patients, independent of concomitant medications, and patients concurrently treated with prednisolone, the AUC ratios were 1.7 and 0.8, respectively. For the POD patient group, the AUC ratio was determined to be 3.2, falling above a twofold discrepancy. The terminal half‐life ratios were between 0.55 and 1.97 for the three patient groupings. The predictive performance of the simulation is shown in Table 7 with MAPE% values of 28.5%, 24.8%, and 60.2% for all patients, prednisolone‐treated, and POD‐treated, respectively. MPE% values were positive for only the prednisolone treatment group and negative for all and POD‐treated patient groups, indicating an over‐prediction of the model for the latter two groups.

**TABLE 5:** Pharmacokinetic parameters from canine observed data and PBPK model simulations with indicated concomitant medications.

Group	AUC_t_ (h × nM)^a^	t_1/2_ ^b^	AUC_t_ Ratio^c^	t_1/2_ Ratio^c^
Observed all^d^	90.51	18.55	1.66	0.55
Observed prednisolone^e^	185.60	15.69	0.81	0.65
Observed POD^f^	47.69	5.15	3.15	1.97
Simulated	150.63	10.15	‐	‐

^a^ AUC_t_ is the area under the concentration–time curve profile from 0 to 24 h.

^b^ t_1/2_ is the terminal half‐life for the elimination as calculated from the linear regression of the terminal elimination phase.

^c^ Ratio is the value generated for corresponding PK parameter from the simulated versus measured dataset.

^d^ All canine patients enrolled in clinical trial regardless of concomitant treatment group.

^e^ Canine patients with concomitant medication of prednisolone only.

^f^ Canine patients with concomitant medication of prednisone/omeprazole/diphenhydramine only.

**FIGURE 4:**
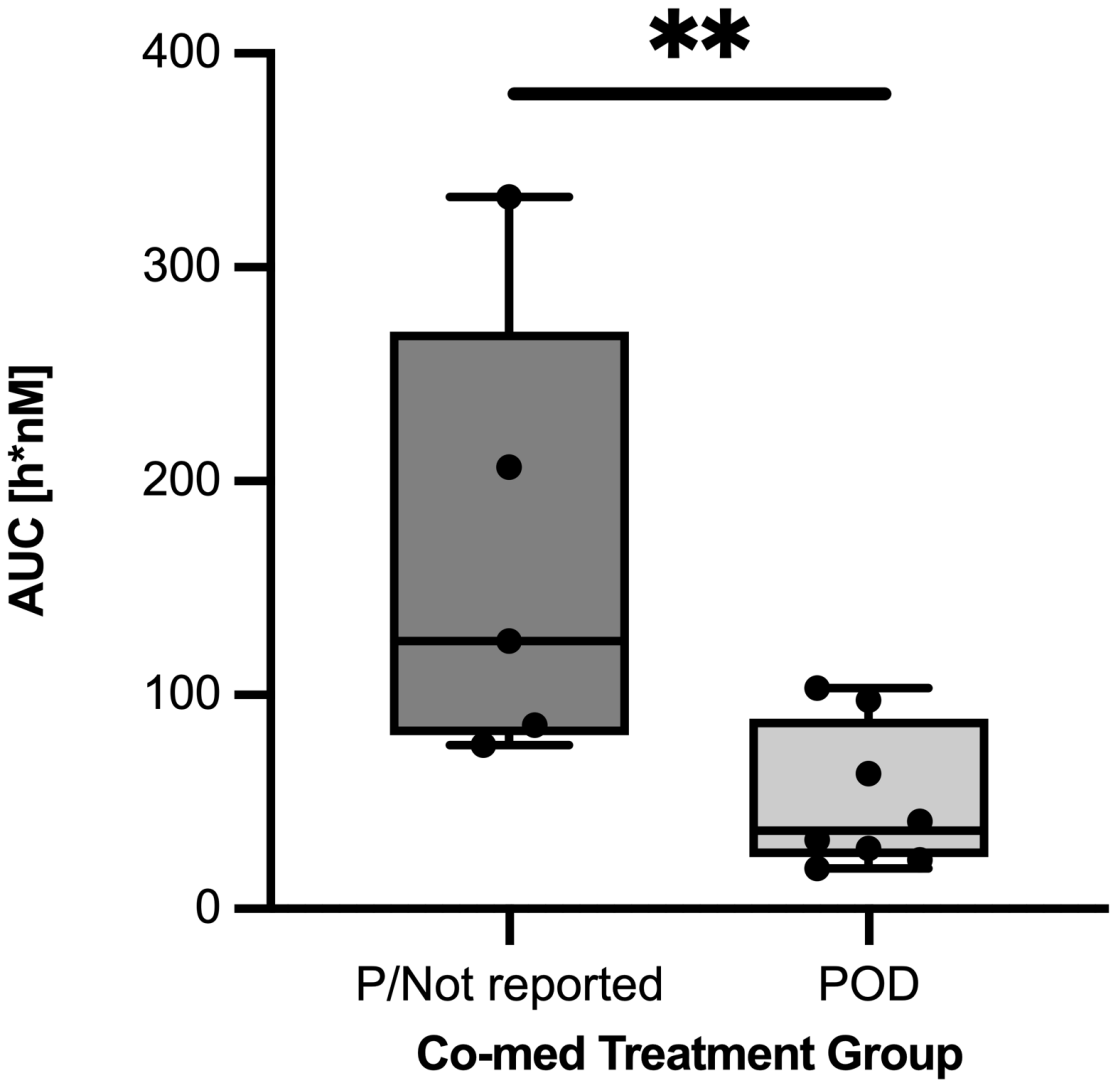
AUC by concomitant medications. Boxplot of AUC values for canine PK respective of concomitant treatment groups shows median ± interquartile range and whiskers represent min/max values; two‐tailed un‐paired t‐test with **p < .01.

Comparison of AUCs for the POD patient group and prednisolone/non‐reported group showed substantially lower values for POD as shown in Figure 4. Omeprazole has been shown in a previous study to be an inducer of CYP3A4 in human HepG2 cells.^41^ Although there are currently no published data indicating the induction of the canine ortholog, CYP3A12, by omeprazole, the potential drug–drug interaction was simulated by increasing the rate of metabolism by twofold, corresponding to the reported induction of CYP3A4 expression.^41^ An analysis of the predictive performance of the escalated metabolism simulation showed improvement in the MAPE% for the POD treatment group from 60.2% to 42.1%.

### 3.3 VBL PK and model simulations in humans

**FIGURE 5:**
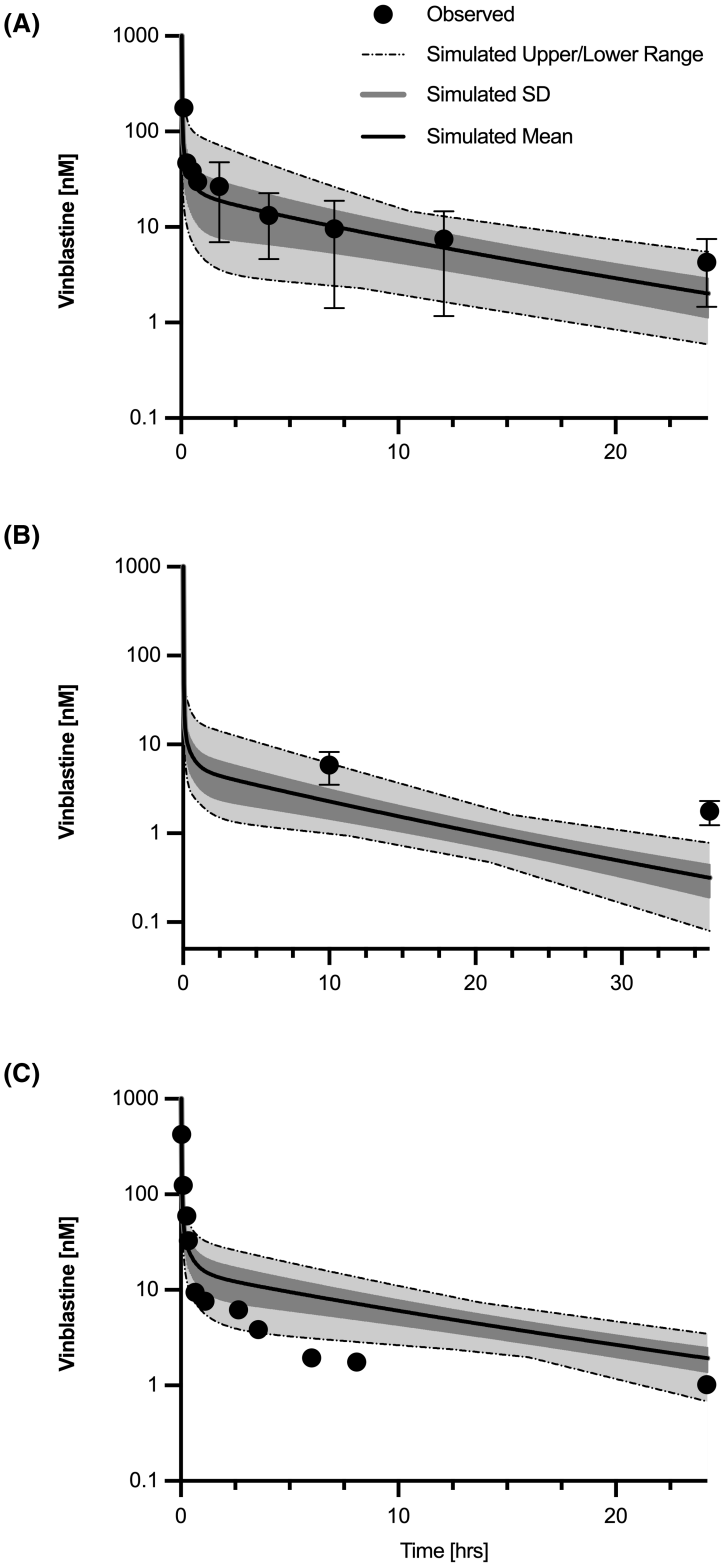
Observed human and Monte Carlo simulated plasma PK. Patients with advanced cancer, mean weight = 43 ± 10.6 kg, mean dose = 0.23 ± 0.08 mg/kg (*n* = 3) (A),.^48^ Patients with non‐small cell lung cancer mean weight = 60.3 ± 10.3 kg, mean dose = 0.083 ± 0.009 mg/kg (*n* = 8),^47^ (B). Patient with metastatic hypernephroma, weight = 59 kg, dose = 0.2 mg/kg (*n* = 1)^21^ (C). Observed Human PK data were extracted from published sources using WebPlotDigitizer.

A human PBPK model was developed using human physiological and clearance parameters as reported in Tables 1 and 3. A Monte Carlo simulation was performed as described for the canine model. Human time course data were sourced from published literature PK profiles for the following IV bolus administered doses: 0.23 ± 0.08, 0.083 ± 0.009, and 0.2 mg/kg^21^, ^47^, ^48^ and digitized using Web Plot Digitizer. Monte Carlo simulations were individually conducted to match corresponding doses and patient weights. Patients undergoing therapy were diagnosed with either advanced, unspecified cancer, non‐small cell lung cancer, or metastatic hypernephroma. The resulting observed and simulated data for the human PBPK model are shown in Figure 5.

PK parameters were estimated from observed and simulated patient data and ratios were calculated for corresponding values. The simulated data for two of the three studies indicated AUC ratios of 0.88 and 1.35 for administered IV bolus doses of 0.23 +/− 0.08 and 0.2 mg/kg. The simulation for an IV bolus dose of 0.083 +/− 0.009 mg/kg for patients concurrently treated with cisplatin was significantly different from observed PK data as the ratio was 0.35. Terminal half‐life ratios were calculated for PK data corresponding to VBL doses of 0.23 +/− 0.08 and 0.2 mg/kg were 0.67 and 0.45, respectively. The observed data for a dose of 0.083 +/− 0.009 mg/kg lacked time course data enabling a terminal half‐life calculation and are thus not reported in Table 6. The accuracy of the prediction was determined by MAPE%, with values of 13.5%, 308.6%, and 58.4%. There was no indicated trend toward a bias with regard tounder‐predicting or over‐predicting the observed patient PK data as the calculated MPE% values were 0.3%, 308.6%, and − 48.8%. One of the three simulations indicated a significant under‐prediction for the simulation corresponding to a mean dose of 0.083 mg/kg. This may be attributed to concurrent IV administered cisplatin and sparse time course data. In summary, the model adequately captured the AUCs for three of the four human PK datasets. AUC values are predominantly underpredicted, though overpredicted for one study,^21^ which highlights the variability in patient PK. The terminal half‐lives for each study were generally underpredicted although one was within a twofold range.

**TABLE 6:** Pharmacokinetic parameters from human observed data and PBPK model simulations.

	Study 1^a^	Study 2^b^	Study 3^c^	Study 4^d^
Dose (mg/kg)	0.23 ± 0.08	0.083 ± 0.009	0.2	0.072 ± 0.04
*n*	3	8	1	24
AUC_t_ (h x nM)^e^				
Obs	239 ± 135	163 ± 62	141	127 ± 66
Sim	211 ± 106	57.8 ± 21.2	191 ± 63	69 ± 26
Ratio	0.88	0.35	1.35	0.55
t_1/2_ (h)^f^				
Obs	10.99	‐	19.7	‐
Sim	7.46	‐	8.78	‐
Ratio	0.67	‐	0.45	‐

^a^ Patients with unspecified advanced cancer treated with IV bolus dose administered VBL.^48^

^b^ Patients with non‐small cell lung cancer treated with IV‐bolus dose administered VBL immediately prior to receiving Cisplatin infusion. Blood samples were collected at 10 and 36 h.^47^

^c^ Patient with metastatic hypernephroma administered IV bolus dose administered VBL.^21^

^d^ Patients with various cancer types (renal, adenoid cystic, prostate, breast, melanoma, sarcoma, Hodgkin's, etc.) treated with IV bolus dose administered VBL. AUC of observed PK was calculated using log trapezoidal method and extrapolated from 0 to infinity. Doses obtained were converted based on a mean patient BSA of 1.67 m^2^ and a mean weight of 70 kg.^49^

^e^ AUC_t_ is the area under the concentration–time curve profile extrapolated for corresponding study time duration and calculated using a linear‐log trapezoidal method for all simulated and observed PK. AUC for simulated PK corresponding to study by Ratain et al.^49^ was extrapolated from 0 to infinity.

^f^ t_1/2_ is the terminal half‐life for the elimination as calculated from the linear regression of the terminal elimination phase.

**TABLE 7:** Measures of predictive performance for PBPK model simulations in mice, canine, and human cancer patients.

Species	MAPE %^a^	MPE%^b^	RMSPE%^c^
Mouse wild‐type			
Serum	42.3	−16.6	97.2
Brain	36.7	−6.4	70.3
Gut	19.3	19.3	30.1
Kidney	14.6	13.6	29.6
Liver	41.9	41.9	53.2
Bone marrow	31.4	−31.4	49.7
Mouse Mdr1a/b(−/−)			
Serum	25.7	−0.3	112.4
Brain	30.0	30.0	105.0
Gut	23.3	22.9	32.1
Kidney	29.3	−4.2	45.1
Liver	23.4	15.2	32.8
Bone marrow	20.	−5.4	49.5
Canine			
All^d^	28.5	−28.5	35.1
Prednisolone^e^	24.8	24.8	73.6
POD^f^	60.2	−60.2	79.8
POD post‐induction^g^	42.1	−42.1	64.
Human			
Nelson 1982	13.5	0.3	100.7
Links et al., 1999	308.6	308.6	172.2
Owellen et al., 1977	58.4	−48.8	307.9

^a^ MAPE% is the median absolute prediction error, which is a measure of the precision of the prediction.

^b^ MPE% is the median prediction error, which is a measure of the bias of the prediction.

^c^ RMSPE% is the root mean squared performance error, which is a measure of the accuracy of the prediction.

^d^ All canine patients enrolled in study independent of concomitant medications.

^e^ Canine patients concomitantly treated with prednisolone only.

^f^ Canine patients concomitantly treated with prednisone, omeprazole, and diphenhydramine.

^g^ Canine model for simulated omeprazole‐induced increase in CYP3A4 expression and metabolic activity for prednisone + omeprazole + diphenhydramine (POD) concomitant treatment group.

## 4 DISCUSSION

Physiologically based pharmacokinetic models were introduced by Teorell in 1937^50^ and first applied to cross‐species modeling of anticancer drugs for methotrexate.^22^ Subsequently, PBPK models have been described for multiple anticancer agents including the cytotoxic drugs, doxorubicin,^51^ docetaxel,^34^ and cisplatin^52^ as well as targeted agents including lapatinib,^53^ crizotinib,^54^ and gefitinib.^55^ A primary component of the predictive utility of these models is the incorporation of attributable processes such as cytochrome P450 metabolism or ATP‐binding cassette transporter transmembrane drug efflux that is isoform specific. Incorporation of these specific enzymatic processes allows for testing of species‐specific parameters, inducers or inhibitors, and polymorphisms altering PK in target populations. Examples include the prediction of exposure to cyclophosphamide and its active metabolite 4‐hydroxycyclophosphamide based on species‐specific K_m_ and V_max_ values for mice, cats, dogs, and humans,^26^ prediction of dose adjustment needed in pediatric patients treated with selumetinib and co‐administered CYP3A4 or CYP2C19 inhibitors or inducers,^56^ and the tissue exposure of doxorubicin in dogs with a polymorphism in ABCB1 leading to a lack of protein expression.^57^

The PBPK model presented here is distinct as it is a first‐generation VBL model that was successfully extrapolated from mouse to canine and human. The predictive model is governed by the well‐established drivers of vinca distribution and elimination: (a) intracellular tubulin binding^2^, ^12^, ^58^ (b) high plasma protein binding, specifically to alpha1‐acid glycoprotein^59^ (c) ABCB1‐mediated transport^16^, ^40^, ^60^ (d) metabolism by isoforms of cytochrome P450 enzyme, CYP3A4,^19^ and CYP3A12^20^ in humans and dogs, respectively, and (e) elimination through glomerular filtration and biliary excretion.^5^ The predominantly flow‐limited PBPK model, consisting of nine distinct compartments, was first developed in wild‐type and Mdr1a/b(−/−) mice. Acquiring VBL PK data in plasma and tissues were critical in model development as it allowed for validation and optimization of parameters that were unobtainable from in vitro experiments. Brain tissue data in Mdr1a/b(−/−) mice were especially important as it permitted the establishment of a permeability‐limited modeling approach to the BBB where VBL passage is uniquely restricted in contrast to other tissues. The model was subsequently extrapolated to different species using species‐dependent physiological and metabolic parameters obtained from in vitro microsomal metabolism experiments. Future work would include quantifying the metabolite, 4‐deacetylvinblastine, in vivo and supplementing the model with its contributing activity. The PBPK model was then coupled to a Monte Carlo simulation to encapsulate relevant patient variability and validated using published observed PK data.

Vinblastine is typically co‐administered with a variety of other medications, either with other cancer therapies in combination protocols or with drugs used to treat other patient maladies. The narrow therapeutic index of VBL warrants an enhanced precision dosing strategy to optimize therapeutic outcomes and minimize associated toxicities. This model provides the quantitative framework to test and validate potential drug–drug interactions and extrapolate to individual physiological conditions such as diminished liver or renal function. An example of such an approach is the recently published PBPK model for vincristine and its application to predict potential drug–drug interactions with Bruton tyrosine kinase inhibitors being added to R‐CHOP protocols for lymphoma.^25^ ABCB1 activity is of particular concern in veterinary medicine as the frequency and variability of canines presenting the ABCB1‐1Δ^61^ mutation necessitates further understanding of its implications in the context of VBL treatment. Although VBL PK levels in all other tissues are not significantly different between the wild‐type and Mdr1a/b(−/−) mouse data, the presence of a known mutation in a clinical setting can have implications on achieved VBL plasma concentrations. Canines known or suspected of having this mutation are often dose reduced, posing a dilemma as it minimizes the risk of toxicity while potentially resulting in inadequate VBL concentrations to achieve therapeutic success. With further assessment of ABCB1 activity in canines and the ability to predictably model VBL distribution, the compromise between appropriate dose reductions and efficacy in chemotherapeutic treatment can be improved. In humans, the role of ABCB1 mutations in VBL dosing has low significance in a clinical setting as there is a lack of reports of major rearrangements of the ABCB1 gene as shown in mice and several dog breeds with varying frequency.^62^, ^63^ The use of P‐gp inhibitors in humans also presents a low risk of increased CNS concentrations of ABCB1 substrates as systemic concentrations do not reach levels to elicit clinically significant ABCB1 transport inhibition.^64^ Additionally, CYP3A enzymes are often a target of induction or repression due to concomitant medication.^65^, ^66^ The dynamic state of key drivers of vinca disposition can thus significantly alter patient‐specific drug clearance. This provides an opportunity for model improvement through supplementation of mRNA expression data for metabolic enzymes and tissue‐specific ABCB1 and tubulin levels in specific patient populations.

The canine cancer patient population that was used for the VBL PK datasets in the studies presented here shows an example of potential drug interactions to be explored with this PBPK model. Dogs receiving omeprazole had a significant decrease in VBL exposure and simulation modeling with altered metabolic activity can identify a VBL dose range that provides an exposure more likely to result in efficacy. Omeprazole is specifically often used to minimize systemic effects of mast cell tumors. Although in some cases the use of an alternative medication for ancillary therapy may suffice, in situations such as the presence of gross disease omeprazole is thought to be more effective than the administration of histamine H1 or H2 blockers such as cimetidine or famotidine.^67^ Therefore, the ability to continue to use a more effective concomitant medication such as omeprazole in specific patient situations can provide therapeutic benefit. The use of these simulations provides the basis and justification for clinical testing of higher doses in these dogs with subsequent PK data to verify or refute the PBPK simulations. The ability to simulate data in special populations and generate testable results allows for further refinement and validation of the model.

In summary, we have successfully developed the first PBPK model for VBL scaled from mice to dogs and humans. The model incorporates VBL‐specific factors including binding to tubulin, CYP3A‐mediated metabolism, and ABCB1‐mediated transport to describe disposition, metabolism, and elimination. The mechanistic framework utilizing key drivers that govern VBL disposition presents a useful tool for providing insight to clinically relevant questions with regard to patient‐specific variability such as co‐morbidities and the effect of drug combinations encountered in therapeutic treatment regimens. An improved understanding of how patient variability affects VBL PK can thus be used to provide predictions in clinical situations and accordingly modify concurrent medications or doses to provide improved treatment to cancer patient populations.

## CONFLICT OF INTEREST

No author has an actual or perceived conflict of interest with the contents of this article.

## Data Availability

The data and modeling code that support the findings of this study are available from the corresponding author upon reasonable request.
